# Electroacupuncture Attenuates Induction of Inflammatory Pain by Regulating Opioid and Adenosine Pathways in Mice

**DOI:** 10.1038/s41598-017-16031-y

**Published:** 2017-11-15

**Authors:** Hsien-Yin Liao, Ching-Liang Hsieh, Chun-Ping Huang, Yi-Wen Lin

**Affiliations:** 1 College of Chinese Medicine, Graduate Institute of Acupuncture Science, China Medical University, Taichung, 40402 Taiwan; 2 Department of Acupuncture, China Medical University Hospital, Taichung, 40402 Taiwan; 3 College of Chinese Medicine, Graduate Institute of Integrated Medicine, China Medical University, Taichung, 40402 Taiwan; 4 Department of Chinese Medicine, China Medical University Hospital, Taichung, 40402 Taiwan; 5 Research Center for Chinese Medicine & Acupuncture, China Medical University, Taichung, 40402 Taiwan

## Abstract

Although inflammatory pain is a common clinical condition, its mechanisms are still unclear. Electroacupuncture (EA), a well-known method of pain management, may reduce inflammatory pain by regulating neurons, astrocytes, and inflammatory signaling pathways. Injections of complete Freund’s adjuvant (CFA), which can initiate cell-mediated inflammatory pain, resulted in significant hyperalgesia, which was subsequently prevented by EA. In CFA-injected mice, a dramatic increase was observed in the expression of the following proteins in the dorsal root ganglion and spinal cord dorsal horn: the astrocytic marker GFAP, S100B, RAGE, pPKCε, COX-2, pERK, and pNFκB. These effects were reversed by EA. In addition, mechanical hyperalgesia was significantly reduced in the N6-cyclopentyladenosine (CPA) i.p. or i.m. and endomorphin (EM) i.p. groups. Neither EM i.m. nor EM i.p. exhibited any analgesic effect on thermal hyperalgesia. However, both CPA i.m. and CPA i.p. attenuated thermal hyperalgesia in the mouse inflammatory pain model. We showed that CPA reduced COX-2 and pPKCε expression. However, EM administration did not reduce COX-2 levels. Combined administration of naloxone and rolofylline increased pPKCε and COX-2 pathways. Taken together, our study results revealed a novel and detailed mechanism of EA-induced analgesia that involves the regulation of the opioid and adenosine pathways.

## Introduction

Inflammatory pain greatly affects the quality of life for countless people worldwide^1^. Despite the numerous side effects of non-steroidal anti-inflammatory drugs, including gastric ulcers, bowel dysfunction caused by morphine, and immune system suppression caused by steroidal drugs, patients spend vast sums of money on these pain medications. Electroacupuncture (EA) is a promising alternative to such drugs and has recently attracted much attention due to increasing evidence of its analgesic effects^2,3^. Previous studies using animal models have demonstrated the therapeutic effects of EA against inflammatory pain via neuronal and non-neuronal pathways, namely suppression of the transient receptor potential cation channel subfamily V member 1 (TRPV1) pathway^4^, generation of anti-nociceptive adenosine on adenosine A1 receptors (A1R) for local acupoints^5–7^, and stimulation of endogenous opioid secretion via the anesthesia pain descending pathway in the central nervous system^8^.

In a previous study, a complete Freund’s adjuvant (CFA) injection into the hind paw of a mouse caused local inflammation and resulted in upstream action potentials toward the spine and central nervous system^9^. Another study has demonstrated that limb inflammation triggers spinal inflammatory activity, with increase in IL-1β, IL-6, TNFα, microglia, and astrocytes levels^10^. Other studies have demonstrated the association of inflammatory pain with various channels and kinases, including TRPV1, voltage-gated sodium channels (VGSC) 1.7 and 1.8, protein kinase A (PKA), protein kinase C (PKC), phosphoinositide 3-kinase (PI3K), serine/threonine kinase, mammalian target of rapamycin, extracellular signal regulated kinase (ERK), cAMP response element-binding protein, and the nuclear factor kappa-light-chain-enhancer of activated B cells (pNFκB)^4,11^. However, to date, details of the mechanisms for spinal inflammatory factors, endogenous opioids, and adenosine remain unclear. Further, very few studies have evaluated how EA may function in these mechanisms to reduce inflammatory pain.

In line with existing reviews, we proposed that PKCε and cyclooxygenase-2 (COX-2) are integral in connecting these pain-related mechanisms. A previous study has indicated that following tissue injury or infection, immune cells secrete inflammatory mediators, such as proinflammatory cytokines, bradykinin, and prostaglandins. These inflammatory mediators act on their respective receptors on peripheral nociceptor neural fibers. The activation of these receptors leads to the generation of secondary messengers, such as Ca^2+^ and cAMP, which in turn activate several kinases (e.g., PKC, PKA, PI3K, and ERK). The activation of these kinases causes hypersensitivity and hyperexcitability of nociceptor neurons via the modulation of key transduction molecules, such as TRPV1 and voltage-gated sodium channels^12^. Interestingly, one study has reported that PKA can switch to PKCε during the transition from early to late phase hyperalgesia^13^.

Researchers also have found that enkephalin activates the presynaptic δ-opioid receptor and inhibits nociceptive VGSC 1.7 in the dorsal root ganglion (DRG) through PKC and p38 inhibition^14^. Both PKA and PKC are involved in the modulation of Nav1.8 currents from neonatal neurons^15,16^. However, the relationship among inflammatory mediators, adenosine, and VGSC remains unclear. One study has revealed that the prostaglandin E2 binds to G-proteins, resulting in a subsequent increase in cyclic AMP levels and consequent activation of PKC signaling pathways and purinergic 2X3 (P2X3) receptors; this ultimately causes exaggerated hyperalgesia^17^. Thus, EA intervention reduces inflammatory pain by suppressing P2X3 receptors as well as activating A1R^5^.

COX-2 is an inflammation-related enzyme that transforms arachidonic acid into different types of prostaglandins, including I2 and E2. These proinflammatory mediators cause inflammation and pain. One study that used an arthritis model has reported that COX-2 inhibitors can suppress prostaglandin generation and inflammation^18^. Another study has demonstrated that PKCε modulates COX-2 generation and plays an important role in an inflammatory pain model^19^. Prophylactic use of non-steroidal anti-inflammatory drugs has been proven to reduce inflammation in ophthalmic and pancreatic diseases and lead to better recovery^20,21^. Further, it is clinically acknowledged that non-steroidal anti-inflammatory drugs can relieve dysmenorrhea and migraines. However, it is unknown whether early use of acupuncture can prevent pain generation in such conditions. Only a few studies have discussed the potential role of acupuncture as a symptomatic treatment to reduce the frequency of headaches^22,23^. Acupuncture, a technique with its origins in Chinese medicine, has been used for over 3000 years across Asia. It has also been recommended by the WHO as an effective analgesic. The present study aimed to identify the role of EA on inflammatory pain in mice and the effects of EA on the regulation of neurons, astrocytes, and other inflammatory signaling pathways.

## Results

### EA significantly reduced mechanical and thermal hyperalgesia in a mouse inflammatory pain model

**Figure 1:**
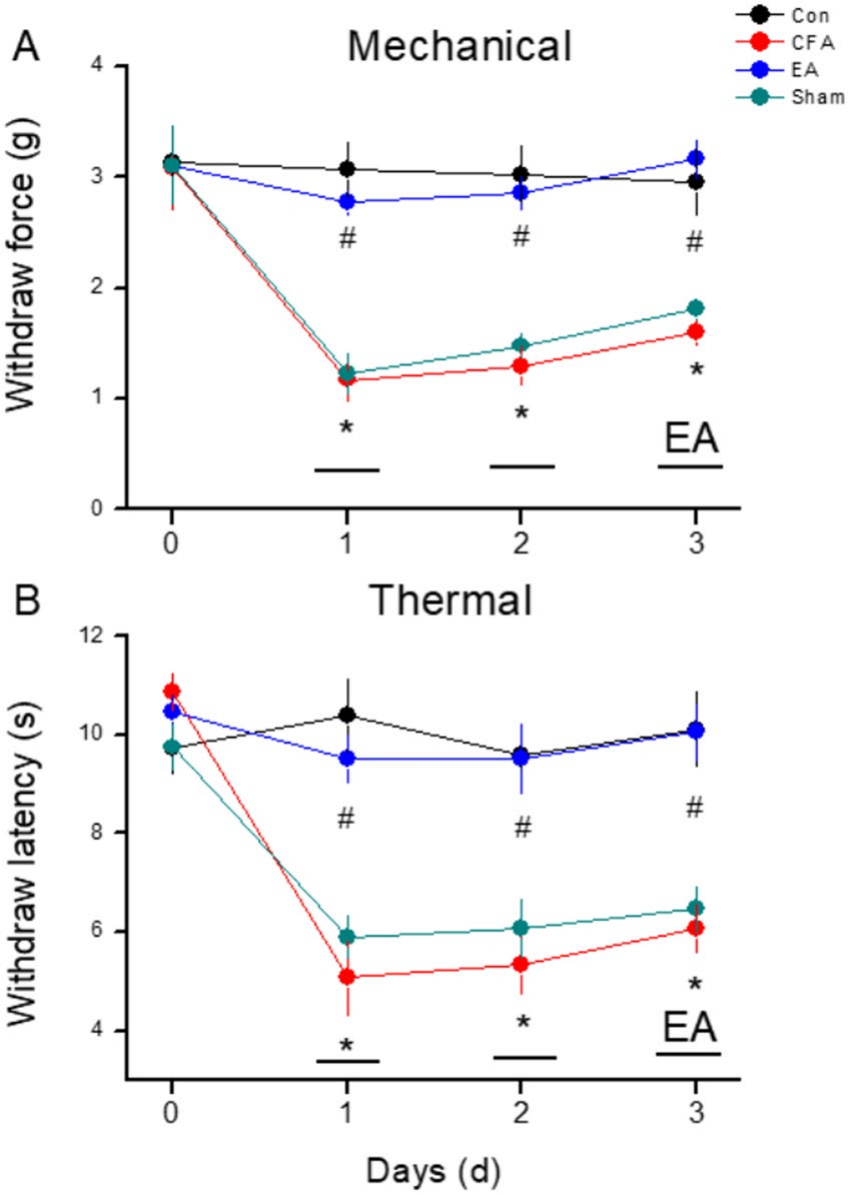
(**A** and **B**). Changes in the withdraw threshold and latency of mice in the von Frey and radial heat test. The picture shows that analgesic effect of EA can be detected on day 1 and day 2 after treatment. *p < 0.05 means CFA compared with control. ^#^ *p* < 0.05 means EA compared with CFA.

As shown in Fig. 1A, no significant difference was observed in mechanical sensitivity under basal conditions among the four groups (Control: 3.14 ± 0.32 g; CFA: 3.08 ± 0.38 g; EA: 3.11 ± 0.29 g; Sham EA: 3.11 ± 0.34 g). In contrast, the pain threshold, also called mechanical hyperalgesia, was significantly lower in the CFA and Sham EA groups on days 1, 2, and 3 after CFA injections (CFA: 1.17 ± 0.19 g, 1.3 ± 0.18 g, and 1.6 ± 0.11 g; Sham EA: 1.23 ± 0.17 g, 1.48 ± 0.10 g, and 1.81 ± 0.09 g, respectively) than in the control group (Control: 3.07 ± 0.25 g, 3.03 ± 0.25 g, and 2.96 ± 0.29 g, respectively). EA significantly prevented the induction of mechanical hyperalgesia (EA: 2.77 ± 0.11 g, 2.86 ± 0.14 g, and 3.16 ± 0.17 g on days 1, 2, and 3, respectively).

As shown in Fig. 1B, no significant difference was observed in withdrawal latencies among the groups prior to CFA injections (Control: 9.74 ± 0.50 s; CFA: 10.88 ± 0.35 s; EA: 10.47 ± 0.32 s; Sham EA: 9.76 ± 0.46 s). However, on days 1, 2, and 3 after CFA injections, withdrawal latencies were shorter in the CFA and Sham EA groups (CFA: 5.07 ± 0.75 s, 5.32 ± 0.56 s, and 6.06 ± 0.48 s; Sham EA: 5.9 ± 0.42 s, 6.07 ± 0.58 s, and 6.46 ± 0.45 s, respectively) than in the control group (Control: 10.41 ± 0.74 s, 9.59 ± 0.45 s, and 10.12 ± 0.76 s, respectively). Interestingly, EA also prevented the induction of thermal hyperalgesia (9.53 ± 0.48 s, 9.51 ± 0.70 s, and 10.06 ± 0.58 s on days 1, 2, and 3, respectively).

### EA reduced non-neuronal and neuronal signaling pathways in the DRG of a mouse inflammatory pain model

**Figure 2:**
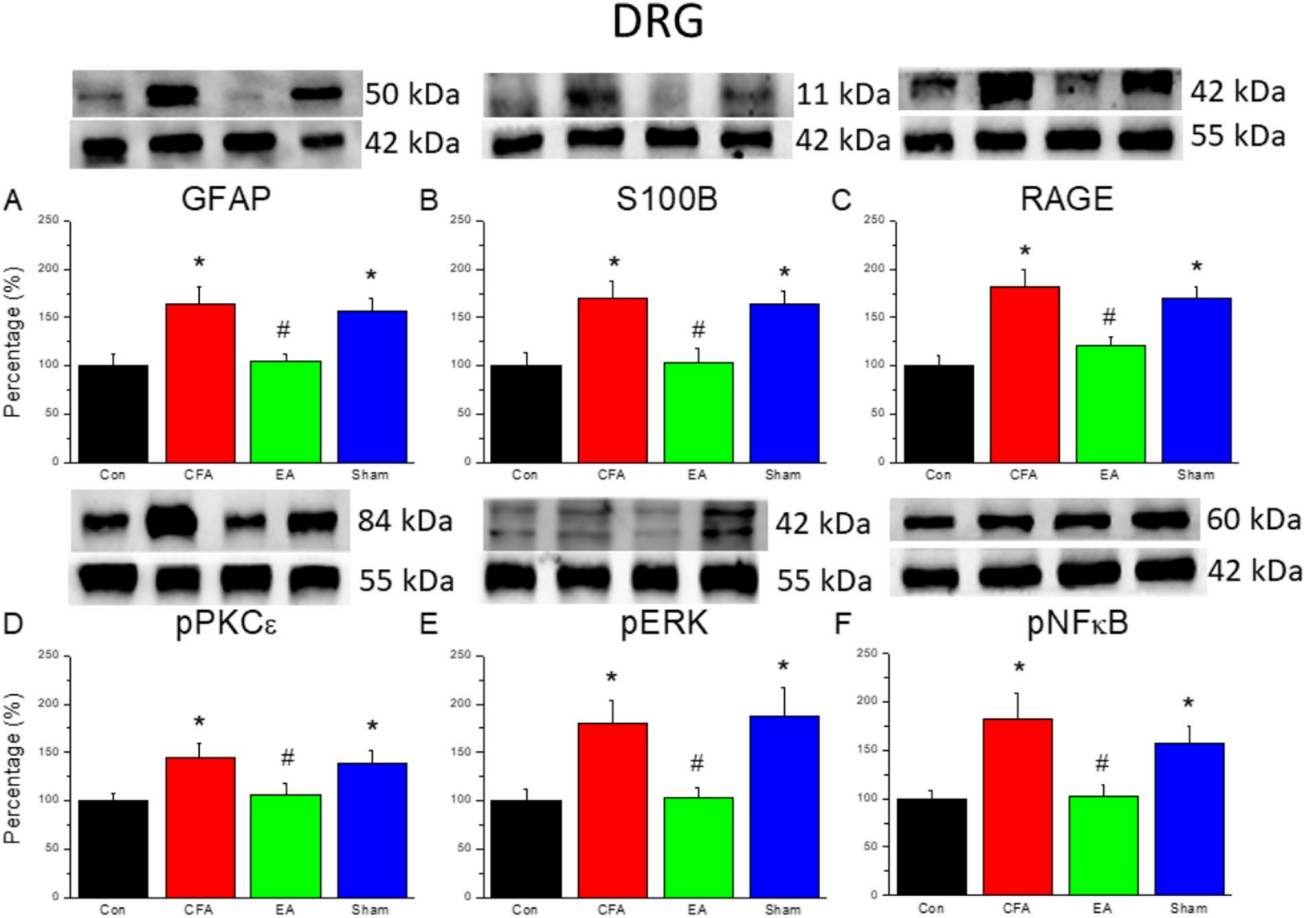
(**A**~**F**) Expression levels from biomarkers of glia cells and inflammatory kinases in DRG aſter CFA injection, EA and Sham EA treatment. *p < 0.05 means CFA or Sham EA compared with Control; ^#^p < 0.05 means EA compared with CFA. The western blot bands at the top show the cropped target protein. The lower bands are cropped internal controls (β-actin or α-tubulin).

As shown in Fig. 2, the levels of inflammatory biomarkers, namely GFAP (an astrocyte cell marker), S100B, and RAGE, around the DRG were upregulated after CFA injections (163.33% ± 17.83%, 170.26% ± 17.75%, and 182.52% ± 17.34%, respectively); EA attenuated these inflammatory biomarkers (104.04% ± 8.93%, 1103.14% ± 14.34%, and 121.11% ± 8.31%, respectively). Similar results were observed for the levels of inflammatory kinases, namely pPKCε, pERK, and pNF-κB, within the DRG (CFA group: 144.62 ± 14.66%, 180.44 ± 23.84%, and 182.87 ± 26.34%; EA group: 105.95 ± 11.90%, 102.96 ± 10.97%, and 102.75 ± 11.29%, respectively).

### EA reduced non-neuronal and neuronal signaling pathways in the spinal cord of a mouse inflammatory pain model

**Figure 3:**
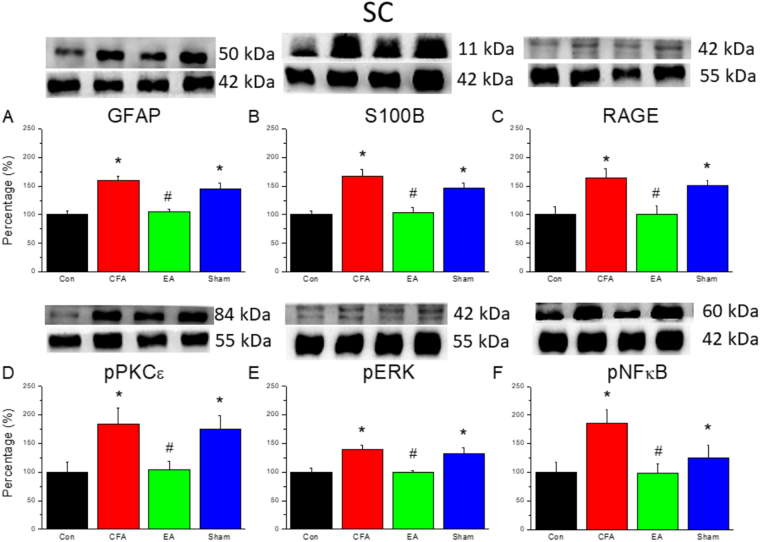
(**A**~**F**) Expression levels from biomarkers of glia cells and inflammatory kinases in DRG aſter CFA injection, EA and Sham EA treatment. *p < 0.05 means CFA or Sham EA compared with Control; ^#^p < 0.05 means EA compared with CFA. The western blot bands at the top show the cropped target protein. The lower bands are cropped internal controls (β-actin or α-tubulin).

As shown in Fig. 3, levels of inflammatory biomarkers, namely GFAP, S100B, and RAGE, around the spinal cord were upregulated after CFA injections (159.12 ± 7.82%, 167.24 ± 12.07%, and 164.87 ± 15.15%, respectively). Importantly, EA attenuated these inflammatory biomarkers (105.18 ± 4.48%, 104.29 ± 8.21%, 100.31 ± 15.10%, respectively). Similar results were observed for levels of inflammatory kinases, namely pPKCε, pERK, and pNF-κB, within the spinal cord (CFA group: 183.46 ± 29.12%, 139.15 ± 7.38%, and 185.57 ± 24.20%; EA group: 104.31 ± 14.35%, 99.28 ± 3.38%, and 98.10 ± 16.83%, respectively).

### Opioid and adenosine A1 receptors play a crucial role in EA-induced analgesia in a mouse inflammatory pain model

**Figure 4:**
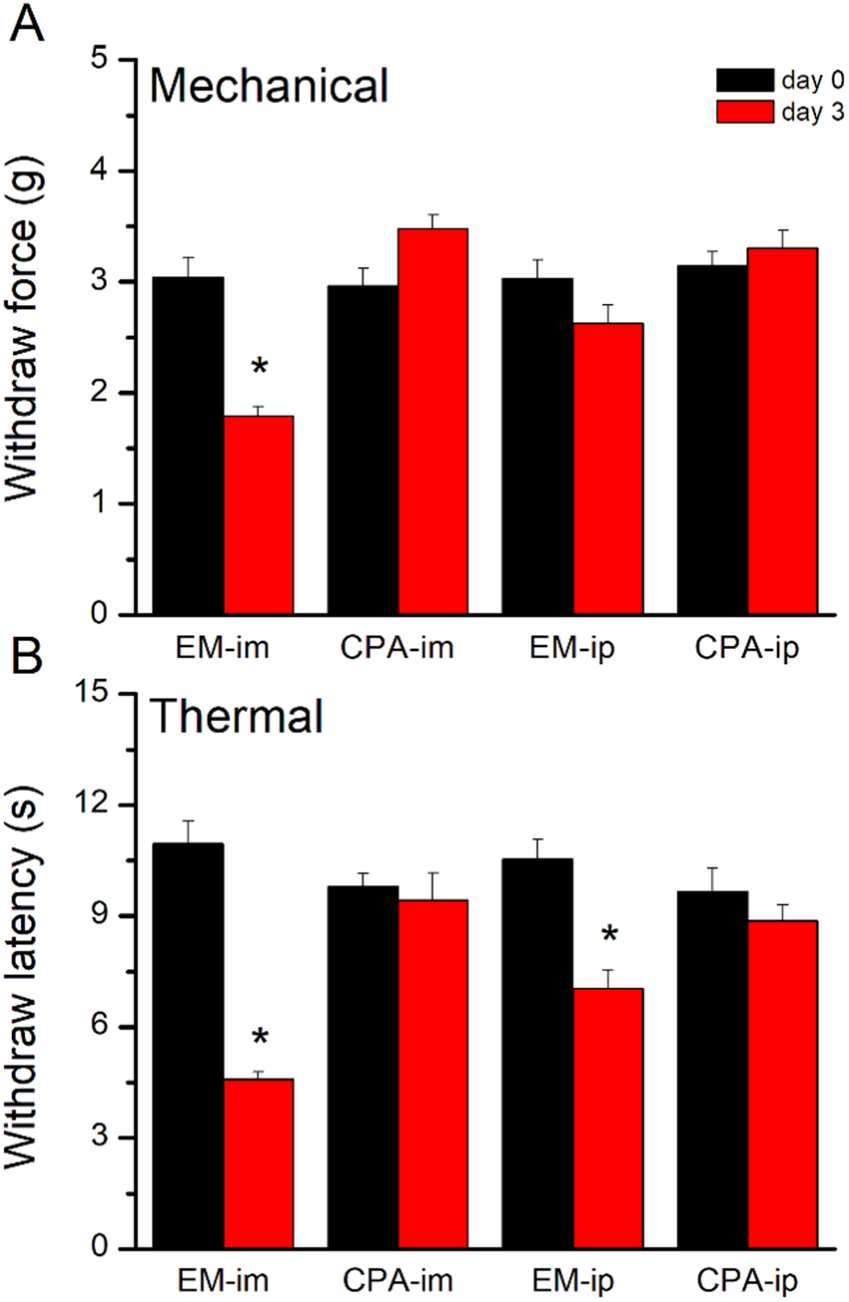
Opioid and adenosine A1 receptor agonist administration relieved mechanical and thermal pain. EM-im: endomorphin intramuscularly injection; CPA-im: N6-Cyclopentyladenosine intramuscularly injection. EM-ip: endomorphin intraperitoneally injection; CPA-ip: N6-Cyclopentyladenosine intraperitoneally injection. *p < 0.05 means comparison with day 0.

Next, to identify the analgesic effects, we used intraperitoneal (i.p.) or intramuscular (i.m.) injections (i.e., acupoint injection) of endomorphin (EM), a μ-opioid receptor agonist, or N6-cyclopentyladenosine (CPA), an adenosine A1 receptor agonist. Mechanical hyperalgesia persisted in the EM i.m. group (Fig. 4A; 1.79 ± 0.09 g, *p* < 0.05 compared with day 0; n = 8). However, mechanical hyperalgesia was significantly reduced in the CPA i.m., EM i.p., and CPA i.p. groups (Fig. 4A; 3.48 ± 0.12 g, 2.63 ± 0.16, and 3.3 ± 0.16 g, respectively; p > 0.05 compared with day 0; n = 8). Furthermore, thermal hyperalgesia was not affected in the EM i.m. and EM i.p. groups (Fig. 4B; 4.58 ± 0.2 s and 7.03 ± 0.51 s, respectively; *p* < 0.05 compared with day 0; n = 8). However, thermal hyperalgesia was attenuated in the CPA i.m. and CPA i.p. groups (Fig. 4B; 9.43 ± 0.72 s and 8.87 ± 0.42 s, respectively; *p* < 0.05 compared with day 0; n = 8).

### Attenuation of CFA-induced nociceptive pPKCε and COX-2 levels by EA, endomorphin, and CPA

**Figure 5:**
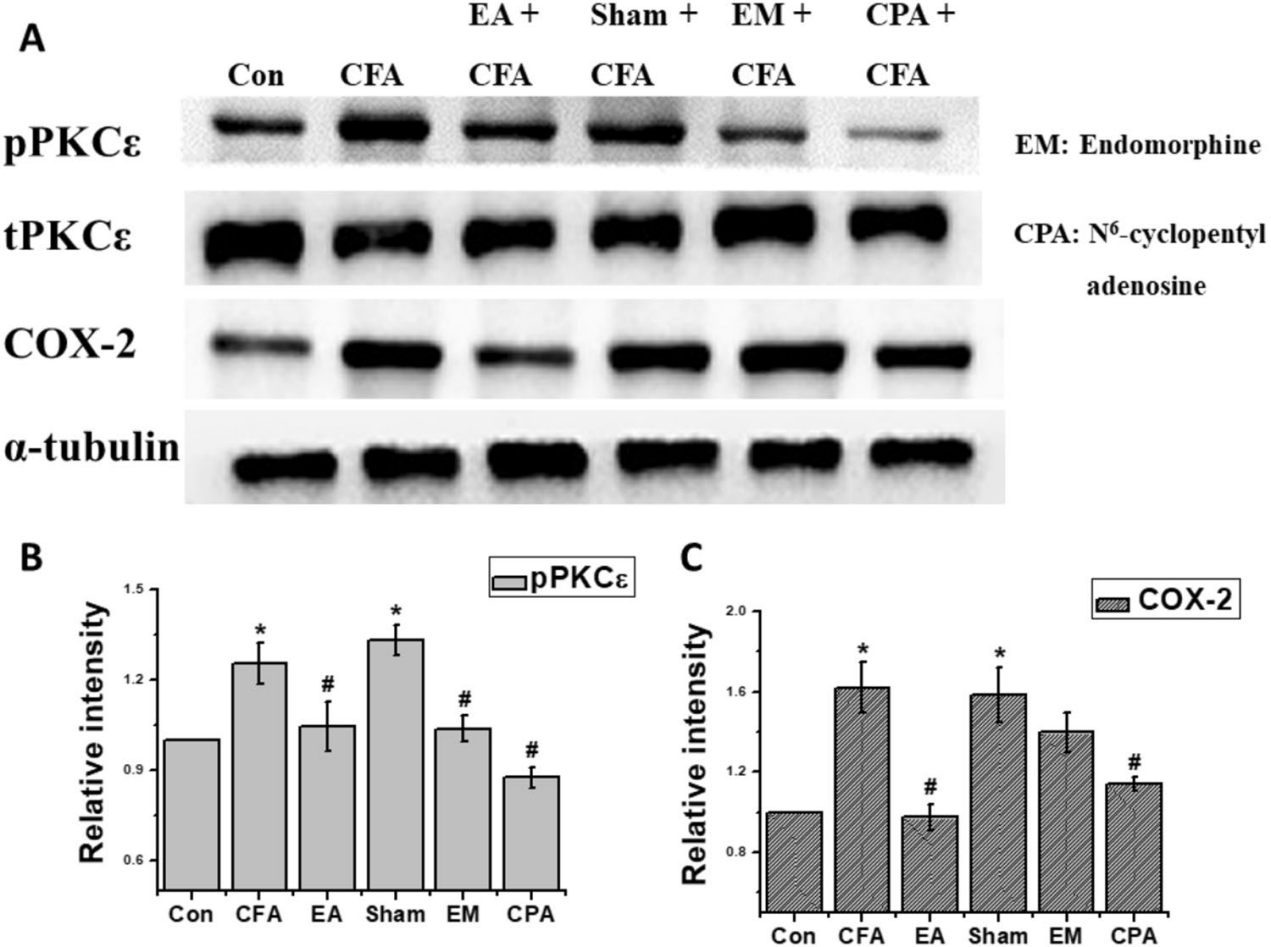
Levels of inflammatory kinases under Electroacupuncture, Sham Electroacupuncture and administration of endomorphin and adenosine agonist. *p < 0.05 means CFA or Sham EA compared with Control; ^#^ *p* < 0.05 means EA, EM and CPA compared with CFA. The western blot bands at the top show the cropped target protein. The lower bands are cropped internal controls (β-actin or α-tubulin).

As shown in Fig. 5A, pPKCε levels within the DRG increased in both the CFA (1.25 ± 0.07) and Sham EA (1.33 ± 0.05) groups. This increase was attenuated by EA (1.05 ± 0.08), EM (1.04 ± 0.04), and CPA (0.88 ± 0.03) administration. In addition, COX-2 levels within the DRG increased in both the CFA (1.62 ± 0.13) and Sham EA (1.58 ± 0.13) groups. This increase was attenuated by EA (0.98 ± 0.07) and CPA (1.14 ± 0.03) administration. Interestingly, EM administration induced lower, although insignificant, COX-2 levels (1.40 ± 0.10) than CFA administration. These results are analyzed and plotted in Fig. 5B and C.

### Combined MOR and A1AR antagonists suppressed the anti-inflammatory effects of EA

**Figure 6:**
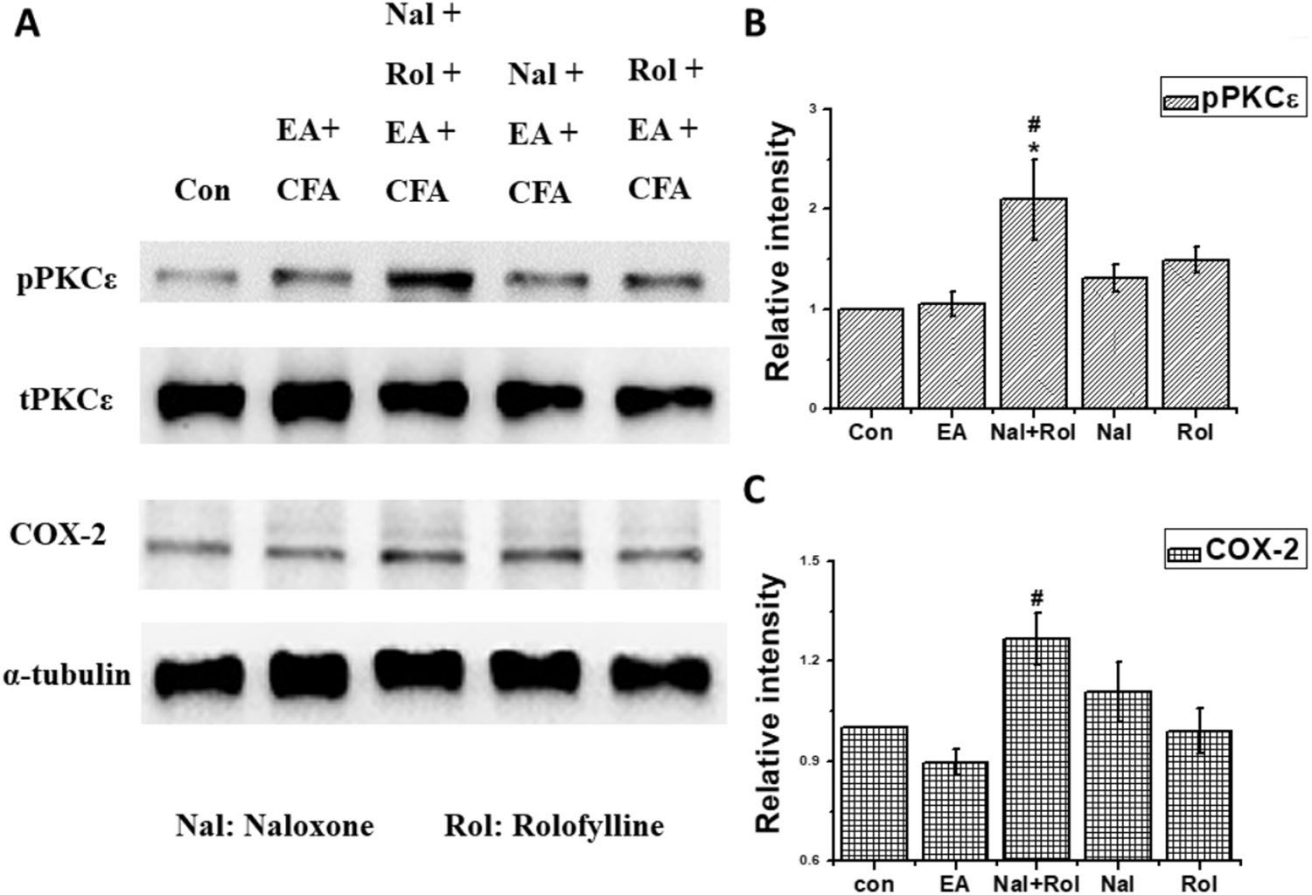
levels of Inflammatory kinases under Electroacupuncture, administration of endomorphin antagonist, adenosine antagonist and combination *p < 0.05 means CFA or Sham EA compared with Control; ^#^ *p* < 0.05 means EA, EM and CPA compared with CFA. The western blot bands at the top show the cropped target protein. The lower bands are cropped internal controls (β-actin or α-tubulin).

**Figure 7:**
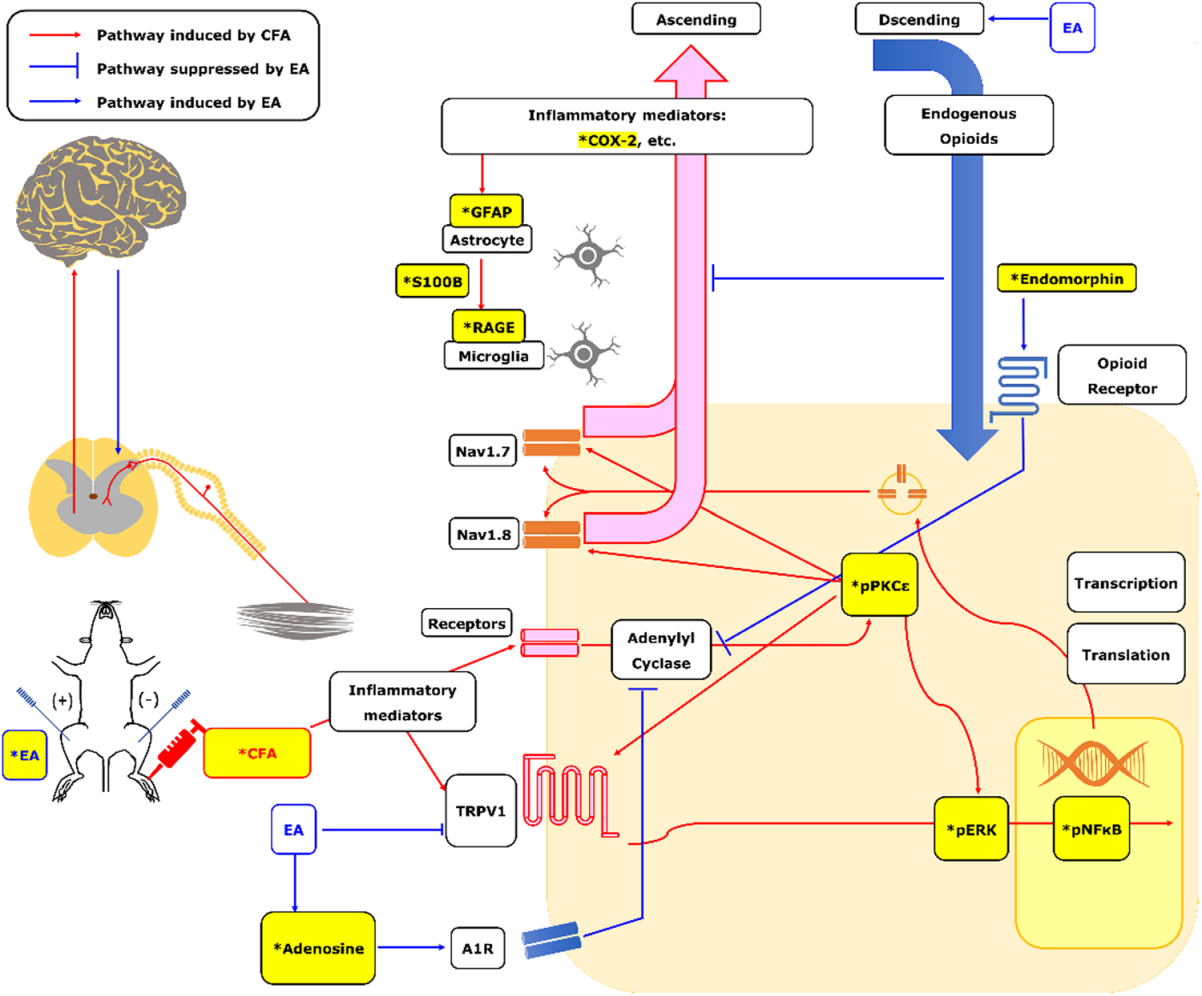
Schematic diagram of possible mechanism in EA-mediated analgesia of CFA-induced inflammatory pain. The contents marked in yellow and * means procedure or factors used in this study. Others are plotted according to previous studies reviewed in introduction.

As shown in Fig. 6A, based on pPKCε levels, both naloxone methiodide (Nal) and rolofylline (Rol) failed to completely block the anti-inflammatory effects of EA (EA: 1.05 ± 0.12, Nal: 1.31 ± 0.13, and Rol: 1.49 ± 0.13). However, combined administration of Nal and Rol significantly increased pPKCε levels (2.09 ± 0.40) compared with EA. Similar results were found for COX-2 levels (also shown in Fig. 6A). COX-2 levels after EA, Nal+Rol, Nal, and Rol administrations were 0.90 ± 0.04, 1.27 ± 0.08, 1.11 ± 0.09, and 0.99 ± 0.07, respectively. All results were analyzed and plotted (Fig. 6B and C). A schematic diagram (Fig. 7) was prepared, and an inflammatory model was designed to determine the relationships among EA, endogenous opioids, adenosine, and inflammatory mediators. Whether EA could attenuate the CFA-induced inflammatory pain was also analyzed.

## Discussion

Despite growing interest in EA and its widespread use in treating dysmenorrhea and migraines, previous research has only partially indicated the mechanisms by which EA suppresses inflammatory pain; only one relatively complete diagram, reported by Lao *et al*. in 2014^8^, suggests a relationship among endogenous opioids, inflammatory cytokines, and adenosine. Thus, the current study aimed to continue our previous research in the context of EA using serial pain models. A research summary is presented in Fig. 7.

As shown in Fig. 1, mechanical and thermal hyperalgesia confirmed that CFA injections successfully evoked inflammatory pain. Next, we demonstrated that EA prevented the induction of mechanical and thermal hyperalgesia on days 1, 2, and 3. pPKCε and COX-2 levels among EA groups also provided indirect evidence that EA attenuated the induction of inflammatory pain, as shown in Fig. 5.

Within the CFA group, we observed an increase in pPKCε, pERK, and pNFκB levels within the DRG and SCDH on day 3 after CFA injections (Figs 2 and 3). The activation of these kinases increased action potentials toward the central nervous system and subsequently resulted in an increase in GFAP expression. Higher GFAP levels suggested that activated astrocytes secrete S100B to stimulate RAGE expression, thereby activating neurons and microglia. In addition, pPKCε, pERK, and pNFκB stimulated proinflammatory cytokines in the spinal cord. A previous study using a rat model of monoarthritis demonstrated IL-1, IL-6, and TNF-α activation following an intra-articular CFA injection^10^. In addition, our study demonstrated that COX-2 levels were stimulated in the DRG.

Administration of the adenosine agonist CPA was found to improve animal pain behaviors and downregulate the expression of COX-2 and phosphorylated PKCε (Fig. 5). However, as shown in Fig. 5, it is unclear why EM administration induced lower, although insignificant, COX-2 levels than CFA administration. One potential explanation is that there was more than one endorphin working in the descending pain suppression pathway at the spinal level. According to a study by Han *et al*. in 2003^24^, four endogenous opioids (namely β-endorphin, enkephalin, endomorphin, and dynorphin) are generated in the central nervous system. During EA, β-endorphin is primarily generated around the periaqueductal gray matter at 2 and 15 Hz, whereas enkephalin and endomorphin are primarily generated around the spinal cord at 2 Hz. Dynorphin is also generated around the spinal cord but at 128 Hz during EA. Therefore, under the 2-Hz EA used in the present study, the endomorphin agonist group could not cover the entire endogenous opioids pathway and thus demonstrated reduced, although insignificant, COX-2 levels compared with those in the CFA group.

## Conclusions

Comparison of the EA and Sham EA groups in this study highlighted the anesthetic specificity of acupoints that non-acupoints lack. Further, by administering the adenosine agonist at a local acupoint and using an i.p. injection of an opioid agonist, we determined the locations at which these drugs affect the pain suppression pathway. Moreover, using an agonist and an antagonist of endogenous opioids and adenosine, we could excite both the adenosine and spinal opioid pathways, demonstrating that EA successfully suppressed inflammatory pain. Blocking the adenosine or opioid pathway alone only partially reduces the anesthetic effects of EA. Furthermore, we demonstrated definitive evidence that EA controls inflammatory factors within the DRG via the two aforementioned pathways, thereby supporting the therapeutic potential of EA treatment for attenuating the induction of inflammatory pain.

## Methods

### Experimental Animals

All animals were treated according to the guidelines of the National Institutes of Health “Guide for the Care and Use of Laboratory Animals,” and the study protocol was approved by the ethics committee of the China Medical University, Taichung, Taiwan (permit No. 2016-061). C57/B6 mice weighing approximately 22–25 g and aged 8–12 weeks were purchased from the BioLASCO Animal Center (Taipei, Taiwan). Animals were housed in plexiglas cages in a temperature-controlled room (25 °C ± 2 °C) with a relative humidity of 60% ± 5% and were fed a diet consisting of standard rat chow and water ad libitum.

### Inflammatory Pain Model

Based on results of our previous studies^4^, it was suggested that a total of 10 mice in each group is the minimum number necessary to perform the experiments. All experiments were performed in our laboratory during daylight hours. First, C57/B6 mice were randomly divided (using simple random sampling) into four groups and then anesthetized with 1% isoflurane for CFA injections and EA treatments. Next, mice were injected with either 20 μl saline (pH 7.4, buffered with 20 mM HEPES) or 0.5 mg/ml CFA (heat-killed M. tuberculosis [Sigma, St. Louis, MO]) in the plantar surface of the hind paw to induce intraplantar inflammation. The four groups were as follows: (1) Control group: anesthesia with normal saline injection, (2) CFA group: anesthesia with CFA injection to induce inflammatory pain, (3) EA group: anesthesia with CFA injection and EA manipulation, and (4) Sham EA: anesthesia with CFA injection and Sham EA, to test the potential impact of acupoint and manipulation.

### Electroacupuncture and Sham Electroacupuncture

EA was conducted in the morning (9:00–10:00 am), immediately after the induction of anesthesia and CFA injections for a total of 15 min, at a frequency of 2 Hz, and at an amplitude of 1 mA. EA procedure was repeated two more times, at 24 and 48 h after CFA injection. The ST36 acupuncture point is located on the tibialis anterior muscle, approximately 1/6 of the distance from the patella to the lateral malleolus. Disposable needles with a diameter of 0.30 mm and a length of 13 mm (Yu-Kuang Acupuncture Instrument Co., Taiwan) were inserted into the muscle layer at both ST36 acupuncture points to a depth of 2–3 mm. Electrical stimulation was produced by a Trio 300 electrical stimulator (Grand Medical Instrument CO., LTD). A similar protocol was used for the nonacupoint (i.e., the upper lateral gluteal muscle, but not the GB30 acupoint) for the sham control group.

### Opioid or Adenosine A1 Receptor Agonist and Antagonist Administration

Adult C57BL/6 male mice (n = 10), aged 8 to 12 weeks, were used in opioid and adenosine agonist analysis. After inflammation was induced, as described above, the μ-opioid agonist EM (Sigma, St. Louis, MO, USA; in 100 µl of saline) was administered i.p. or i.m. at a dose of 10 mg/kg, once per day. Alternatively, the adenosine receptor agonist CPA (Sigma, St. Louis, MO, USA; in 10 µl of saline) was immediately administered i.p. or i.m. into acupoint ST36 at a dose of 0.1 mg/kg, once per day. Under light isoflurane anesthesia (1%), EM and CPA were given 24 h after CFA injection.

The other group of adult C57BL/6 male mice (n = 10), aged 8 to 12 weeks, were used for opioid and adenosine *antagonist* analysis. The opioid antagonist Nal (Sigma, St. Louis, MO, USA; in 100 µl of saline) was injected i.p. at a dose of 10 mg/kg. The adenosine A1 receptor antagonist Rol (Sigma, St. Louis, MO, USA; in 10 µl of saline) was injected i.m. at a dose of 3 mg/kg into acupoint ST36. Nal, Rol, and a combination of Nal and Rol were given immediately following CFA injection and were then followed by EA treatments.

### Behavior Test (von Frey test and Hargraves’ test)

By using behavior tests at the moment after CFA injection, 24, 48, and 72 hours later, we checked whether there were mechanical and thermal hyperalgesia. All stimuli were administered at room temperature (approximately 25 °C) and applied only when the animals were calm, but not sleeping or grooming. Mechanical sensitivity was measured by testing the force of responses to stimulation with three applications of electronic von Frey filaments (North Coast Medical, Gilroy, CA, USA). Thermal pain was measured with three applications using Hargraves’ test IITC analgesiometer (IITC Life Sciences, SERIES8, Model 390 G).

### Tissue sampling and Western blot analysis

Mice aged 8–12 weeks were sacrificed by use of CO2 to minimize their suffering. L3-L5 DRG and SCDH were harvested three days later after injection of CFA and then immediately excised to extract proteins. Total proteins were prepared by homogenized DRG and SCDH in lysis buffer containing 50 mM Tris-HCl pH 7.4, 250 mM NaCl, 1% NP-40, 5 mM EDTA, 50 mM NaF, 1 mM Na3VO4, 0.02% NaN3 and 1× protease inhibitor cocktail (AMRESCO). The extracted proteins (30 μg per sample assessed by BCA protein assay) were subjected to 8% SDS-Tris glycine gel electrophoresis and transferred to a PVDF membrane. The membrane was blocked with 5% nonfat milk in TBS-T buffer (10 mM Tris pH 7.5, 100 mM NaCl, 0.1% Tween 20), incubated with first antibody (anti-GFAP, anti-s100B, anti-RAGE, anti-pPKCε, anti-pERK, anti-pNF-κB, anti-COX-2) in TBS-T with 1% bovine serum albumin, and incubated for 1 hour at room temperature. Peroxidase-conjugated anti-rabbit antibody (1:5000) was used as a secondary antibody. The bands were visualized by an enhanced chemiluminescencent substrate kit (PIERCE) with LAS-3000 Fujifilm (Fuji Photo Film Co. Ltd). Where applicable, the image intensities of specific bands were quantified with NIH ImageJ software (Bethesda, MD, USA). Images were gathered in to figures by using photoimpact 12 without any unnecessary edition.

### Statistical Analysis

All statistic data were presented as the mean ± standard error. A *P* value < 0.05 was considered to represent statistical significance. The four groups in this study were: (1) Control group, (2) CFA group, (3) EA group, and (4) Sham EA group (n = 10/group). Statistical significance between groups was tested using the ANOVA test, followed by a post hoc Tukey’s test (*p* < 0.05 was considered statistically significant). All statistical analyses were carried out using the statistical package SPSS for Windows (Version 21.0, SPSS, Chicago, Illinois, USA).
